# Generative pre-trained transformer 4o (GPT-4o) in solving text-based multiple response questions for European Diploma in Radiology (EDiR): a comparative study with radiologists

**DOI:** 10.1186/s13244-025-01941-7

**Published:** 2025-03-22

**Authors:** Jakub Pristoupil, Laura Oleaga, Vanesa Junquero, Cristina Merino, Ozbek Suha Sureyya, Martin Kyncl, Andrea Burgetova, Lukas Lambert

**Affiliations:** 1 Department of Imaging Methods, Motol University Hospital and Second Faculty of Medicine, Charles University, Prague, Czech Republic; 2 Department of Radiology, Clinical Diagnostic Imaging Centre, Hospital Clínic de Barcelona, Barcelona, Spain; 3 Era Radiology Center, Izmir, Turkey; 4 Department of Radiology, First Faculty of Medicine, Charles University and General University Hospital in Prague, Prague, Czech Republic

**Keywords:** Examination, Radiology, Artificial intelligence, Natural language processing

## Abstract

**Objectives:**

This study aims to assess the accuracy of generative pre-trained transformer 4o (GPT-4o) in answering multiple response questions from the European Diploma in Radiology (EDiR) examination, comparing its performance to that of human candidates.

**Materials and methods:**

Results from 42 EDiR candidates across Europe were compared to those from 26 fourth-year medical students who answered exclusively using the ChatGPT-4o in a prospective study (October 2024). The challenge consisted of 52 recall or understanding-based EDiR multiple-response questions, all without visual inputs.

**Results:**

The GPT-4o achieved a mean score of 82.1 ± 3.0%, significantly outperforming the EDiR candidates with 49.4 ± 10.5% (*p* < 0.0001). In particular, chatGPT-4o demonstrated higher true positive rates while maintaining lower false positive rates compared to EDiR candidates, with a higher accuracy rate in all radiology subspecialties (*p* < 0.0001) except informatics (*p* = 0.20). There was near-perfect agreement between GPT-4 responses (κ = 0.872) and moderate agreement among EDiR participants (κ = 0.334). Exit surveys revealed that all participants used the copy-and-paste feature, and 73% submitted additional questions to clarify responses.

**Conclusions:**

GPT-4o significantly outperformed human candidates in low-order, text-based EDiR multiple-response questions, demonstrating higher accuracy and reliability. These results highlight GPT-4o’s potential in answering text-based radiology questions. Further research is necessary to investigate its performance across different question formats and candidate populations to ensure broader applicability and reliability.

**Critical relevance statement:**

GPT-4o significantly outperforms human candidates in factual radiology text-based questions in the EDiR, excelling especially in identifying correct responses, with a higher accuracy rate compared to radiologists.

**Key Points:**

- In EDiR text-based questions, ChatGPT-4o scored higher (82%) than EDiR participants (49%).
- Compared to radiologists, GPT-4o excelled in identifying correct responses.
- GPT-4o responses demonstrated higher agreement (κ = 0.87) compared to EDiR candidates (κ = 0.33).

**Graphical Abstract:**

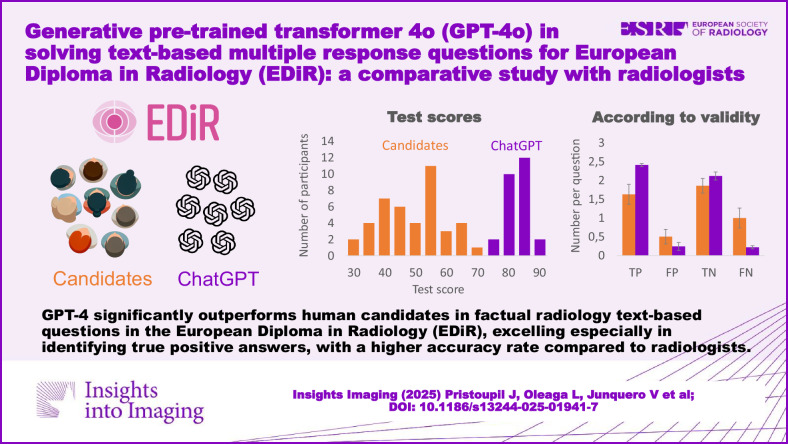

## Introduction

The rapid advancements in the power, accuracy, and versatility of large language models (LLM) such as generative pre-trained transformers (GPT), Gemini, or Copilot have significantly expanded their applications. These models are now capable of performing tasks that not only require vast global knowledge but also involve reasoning and the interpretation of complex relationships with expert-level precision [1]. While LLMs are designed primarily for generating text from input prompts, their potential has been evaluated across various domains, including medical sciences and healthcare, including radiology [2].

The primary function of language models is not the interpretation of visual inputs such as medical images. In radiology, their potential role has been recognized in various areas including the selection of appropriate imaging examinations and protocols, standardization, interpretation, and summarization of radiology reports, error detection, data extraction from reports, generation of differential diagnoses, and radiology education [1, 3–7]. LLMs have also been evaluated in board examinations for medical professionals, including radiologists, showing their superiority in recall-based tasks and, to a lesser extent, in clinical reasoning.

For example, ChatGPT-4 achieved an accuracy of 84% when answering low-order thinking single best answer (SBA) questions that mirrored the style, content, and difficulty of the American Board of Radiology and Canadian Royal College exams. Its performance was notably lower in tasks requiring the application of concepts (30%) or involving calculation and classification (25%), although it nearly achieved a passing grade overall [8]. However, these evaluations did not involve actual exam questions, as the test content was strictly confidential.

The European Diploma in Radiology (EDiR) examination is available to board-certified radiologists and radiology residents in their final year of training [9]. The European Board of Radiology organizes EDiR sessions across multiple continents. EDiR allows candidates to demonstrate their radiological expertise and advance their careers [9]. The examination is conducted in English; approximately 1000 candidates, many of whom are non-native English speakers, participate in the exam annually.

The aim of this study was to conduct a head-to-head comparison between ChatGPT-4 and former EDiR candidates in terms of accuracy when solving text-based questions from the examination.

## Materials and methods

This prospective study was conducted in accordance with the ethical standards outlined in the Declaration of Helsinki. Ethical approval for this study was obtained from the Ethics Committee of the General University Hospital in Prague (79/24 S-IV Grant). Written informed consent, along with general data protection regulation (GDPR) protection and non-disclosure statements, was obtained from all participants prior to their inclusion in the study.

### Study participants

Fourth-year medical students (*n* = 385) were invited via e-mail to participate in this study. The response form was available from October 9 to October 10, 2024, by which time 33 responses were collected from students who met the following inclusion criteria: current enrollment in the fourth year of the general medicine program (before the radiology course), an English proficiency level of upper intermediate English proficiency level (B2) or higher (e.g., First Certificate, state exam, or school-leaving exam), and a grade of A in the Medical English examination.

Data were collected on participants’ English proficiency, year of birth, gender, and prior experience with LLMs. Participants were invited to a ChatGPT challenge for compensation that included 22.2 Euro (EUR) for the ChatGPT-4o upgrade and 17.2 EUR for their time and effort.

### Test questions

The EDiR test session was constructed using questions from official EDiR sessions administered in 2023. Only text-based questions without any visual prompts were selected. The final set consisted of 52 low-order thinking multiple-response questions (MRQs), each with 5 answer options, covering the areas listed in Table 1. The score for each question is calculated as follows:

$${{{{\rm{Score}}}}\;{{{\rm{per}}}}\;{{{\rm{question}}}}}\,= 	 \frac{{{{\rm{correct}}}}\;{{{\rm{answers}}}}}{{{{\rm{number}}}}\;{{{\rm{of}}}}\;{{{\rm{correct}}}}\;{{{\rm{answers}}}}}\\ 	 -\,\frac{{{{\rm{incorrect}}}}\;{{{\rm{answers}}}}}{{{{\rm{number}}}}\;{{{\rm{of}}}}\;{{{\rm{incorrect}}}}\;{{{\rm{answers}}}}}$$

**Table 1:** Question types in the EDiR challenge (ordered alphabetically) and scores achieved by EDiR candidates and ChatGPT

Subspeciality	No	Clinical	Technical	Score		
				Candidates	ChatGPT	*p* value
Abdominal radiology	6	x		52.4 ± 17.6	82.2 ± 7.4	*p* < 0.0001
Breast radiology	3	x		55.7 ± 20.3	97.7 ± 5.4	*p* < 0.0001
Cardiovascular radiology	2	x		49.3 ± 21.3	90.4 ± 12.4	*p* < 0.0001
Emergency medicine	2	x		50.1 ± 17.9	92.3 ± 11.8	*p* < 0.0001
Head and neck imaging	4	x		56.6 ± 19.8	83.4 ± 9.6	*p* < 0.0001
Informatics	2		x	40.5 ± 37.0	50.0 ± 0.0	0.20
Interventional radiology	2	x		61.0 ± 25.8	93.8 ± 8.1	*p* < 0.0001
Management	2		x	50.9 ± 17.1	71.1 ± 9.0	*p* < 0.0001
Musculoskeletal radiology	6	x		53.3 ± 16.0	91.5 ± 4.8	*p* < 0.0001
Neuroimaging	5	x		59.6 ± 19.9	88.2 ± 11.5	*p* < 0.0001
Pediatric radiology	3	x		31.8 ± 18.8	75.5 ± 18.2	*p* < 0.0001
Physics	3		x	38.2 ± 19.5	92.8 ± 4.2	*p* < 0.0001
Pharmacology	2		x	41.9 ± 20.5	69.2 ± 14.2	*p* < 0.0001
Thoracic radiology	6	x		45.8 ± 15.6	68.4 ± 5.5	*p* < 0.0001
Urogenital radiology	4	x		42.0 ± 20.0	77.2 ± 12.5	*p* < 0.0001
Total	52	43	9			

The scores are presented as mean ± standard deviation

The maximum score for a question was 1, with a minimum score of 0, and no negative marking was applied. Marking and correct answers were provided by the administrator. The test score was calculated as follows:

$${{{{\rm{Test}}}}\;{{{\rm{score}}}}}\,=\,\frac{\mathop{\sum }_{i=1}^{52}{{{{\rm{score}}}}\;{{{\rm{per}}}}\;{{{\rm{question}}}}}_{i}}{52}\,\times\,100\%$$

### EDiR testing

The EDiR examinations are conducted at various locations across multiple continents. For this study, we included all 42 candidates who participated in the examination in 2023 at two European locations. No demographic data about the participants were collected to maintain confidentiality. The EDiR examination is open to board-certified radiologists and radiology residents in their final year of training, who are expected to possess comprehensive general knowledge in radiology [9].

### ChatGPT challenge

The ChatGPT challenge took place on October 22^nd^ and 23^rd^, 2024, in a computer room starting at 1 pm. Prior to the test, students were introduced to the use of ChatGPT-4o, including creating prompts, specifying tasks, contexts, personas, and formats, asking detailed questions, and utilizing new prompts. The test was administered in the same web-based environment as that faced by actual EDiR participants, with the exception that copy-paste functionality was enabled and the time limit was set to 180 min. The students were instructed to submit responses based solely on the ChatGPT-4o output, disregarding their own opinions, knowledge, or intuition. Upon completion of the test, the time was recorded, and all the students were further queried about the strategies they employed in generating the ChatGPT prompts.

### Power analysis

A power analysis was performed to determine the sample size needed to compare two independent groups based on mean scores obtained from EDiR candidates. Using a mean score of 0.49, a standard deviation (SD) of 0.10, and an expected difference of 0.1, a sample size of 18 participants would be required to achieve a statistical power of 0.8 and an alpha level of 0.05.

### Statistical analysis

Statistical analyses were conducted using Prism (GraphPad, La Jolla, CA) and R (R Foundation, Vienna, Austria). The data distribution was evaluated using the D’Agostino-Pearson omnibus test and visual inspection of histograms. Grubb’s test was employed to identify outliers. Continuous variables were expressed as mean ± SD. Group differences were analyzed using the *t*-test. Interobserver agreement was calculated as Fleiss kappa (library *irr*) and 95% confidence intervals by bootstrapping (library *boot*). A *p* value < 0.05 was considered statistically significant.

## Results

**Fig. 1:**
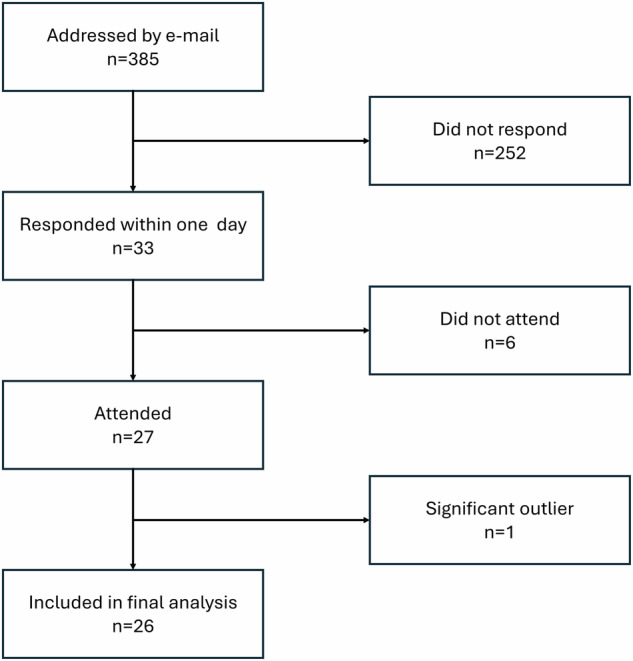
Study flowchart (students)

Among 33 students who completed the initial questionnaire and were invited, 27 ultimately participated in the ChatGPT challenge. One student was excluded as a significant outlier, likely due to erroneously switching to a different ChatGPT model, leaving 26 participants for the final analysis (Fig. 1). Their self-reported English proficiency level was B2 (*n* = 13), advanced English proficiency level (C1) (*n* = 8), or proficient English proficiency level (C2) (*n* = 5). They were born in either 2001 (*n* = 5) or 2002 (*n* = 21), with the group comprising 15 males and 11 females (Table 2). Twenty-one (81%) students reported prior experience with ChatGPT. All but one (96%) had experience with chatbots and 9 (35%) reported frequent usage (Table 2).

**Table 2:** Characteristics of the ChatGPT challenge participants (students)

Item	Number (%)
English Level	
B2	13 (50%)
C1	8 (31%)
C2	5 (19%)
Year of birth	
2001	5 (19%)
2002	21 (81%)
Gender	
Male	15 (58%)
Female	11 (42%)
Chatbot usage	
Never	1 (4%)
Rarely	4 (15%)
Sometimes	12 (46%)
Frequently	9 (35%)
Chatbot usage*	
ChatGPT	21 (81%)
Copilot	5 (19%)
Gemini	3 (12%)
None	4 (15%)

* More answers may be checked

**Fig. 2:**
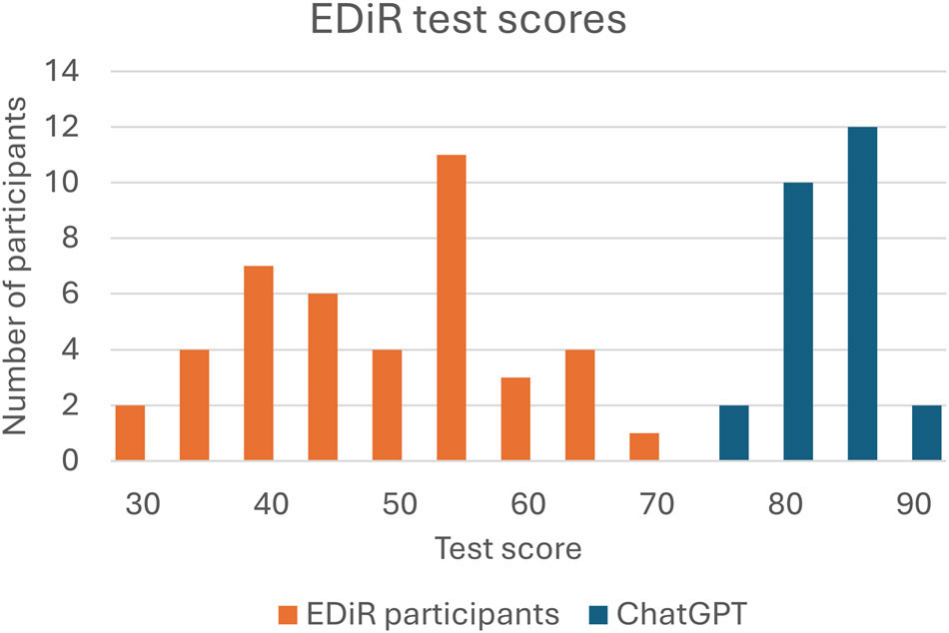
Histogram of test scores. The scores of EDiR candidates (*n* = 42, orange bars) were lower and exhibited greater dispersion compared to those of ChatGPT (*n* = 26, blue bars)

**Fig. 3:**
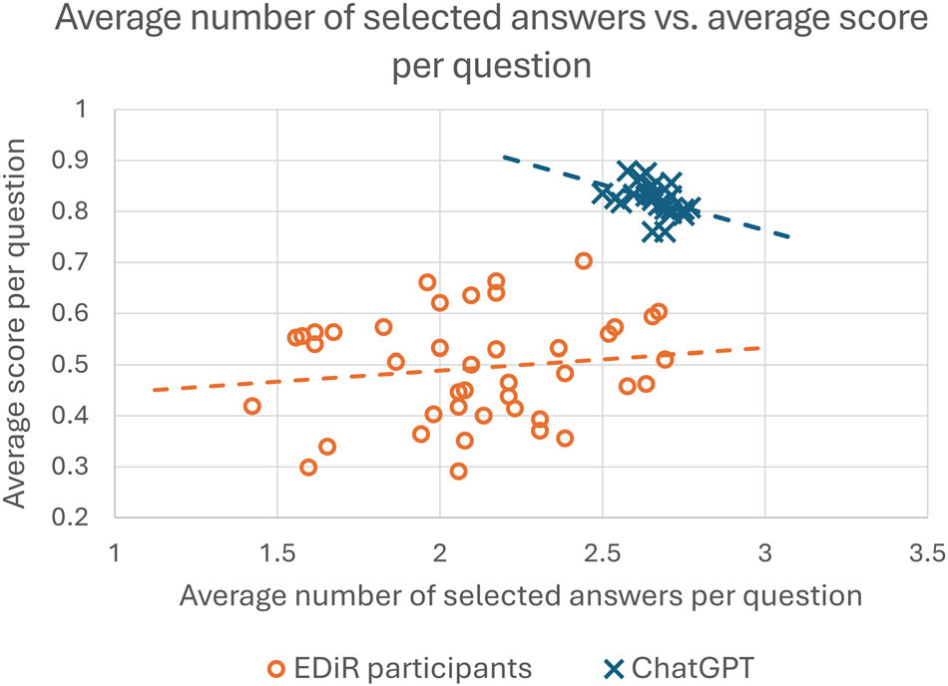
The average number of selected answers vs average score per question

**Fig. 4:**
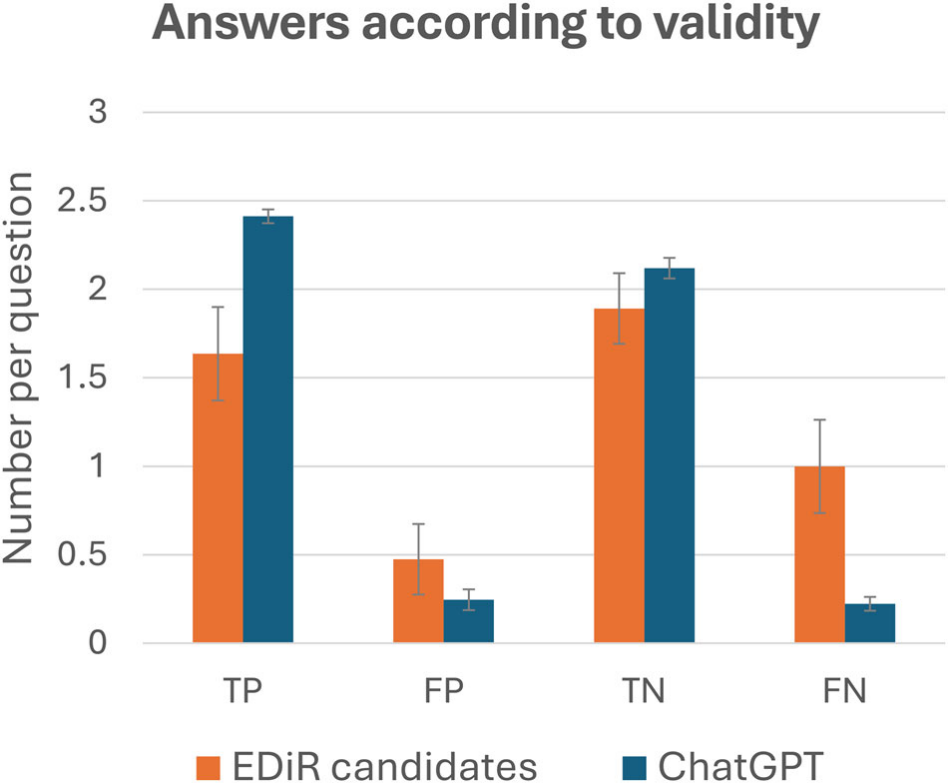
Comparison of the number of answers according to their validity. TP, true positive; FP, false positive; TN, true negative; FN, false negative

The students completed the test in an average time of 60.7 ± 11.7 min, achieving a mean score of 82.1 ± 3.0%, compared to the EDiR candidates, who scored 49.4 ± 10.5% (*p* < 0.0001, Fig. 2). ChatGPT-4o outperformed the EDiR candidates across all subspecialties (*p* < 0.0001), except in informatics (*p* = 0.20, Table 1). The EDiR candidates selected on average 2.11 ± 0.05 answers per question compared to students using ChatGPT-4o with 2.66 ± 0.01 answers (*p* = 0.0001, Fig. 3). ChatGPT-4o achieved higher true positive (TP) rates while maintaining lower false positive (FP) rates compared to EDiR candidates (Fig. 4 and Table 3). The agreement between EDiR candidates was fair (κ = 0.33; 95% CI: 0.29–0.38) compared to the near-perfect agreement between GPT-4’s responses (κ = 0.87; 95% CI: 0.84–0.90).

**Table 3:** Comparison of the number of true positive (TP), false positive (FP), true negative (TN), and false negative (FN) answers between EDiR candidates (radiologists) and ChatGPT

	EDiR candidates	ChatGPT	*p*
TP	1.63 ± 0.27	2.41 ± 0.04	< 0.0001
FP	0.47 ± 0.19	0.25 ± 0.06	< 0.0001
TN	1.89 ± 0.19	2.12 ± 0.06	< 0.0001
FN	1.00 ± 0.27	0.23 ± 0.04	< 0.0001

In the exit survey, all participants reported using the copy-and-paste feature enabled specifically for the challenge. Sixteen participants (62%) added extra prompt information such as persona, setting, or specification of the task. Seventeen participants (65%) regularly used new prompts, and 19 (73%) submitted additional questions for clarification. There was no significant difference in scores between participants who employed additional prompt specifications (*p* = 0.23), used new prompts (*p* = 0.46), or asked additional queries (*p* = 0.67) compared to those who did not (Table 4).

**Table 4:** Exit survey summary

	Yes		No		
	Number (%)	Score	Number (%)	Score	*p* value
Copy and paste	26 (100%)	82.1 ± 3.0	0		
Additional prompt	16 (62%)	81.5 ± 3.1	10 (38%)	83.0 ± 2.6	0.2252
New prompt	17 (65%)	81.7 ± 3.0	9 (35%)	82.7 ± 2.9	0.4552
Additional queries	19 (73%)	82.2 ± 3.3	7 (27%)	81.7 ± 2.1	0.6706

## Discussion

This study demonstrated that ChatGPT-4o significantly outperformed regular participants in low-order, non-visual EDiR questions across all subspecialties, except for informatics. ChatGPT-4o achieved higher TP rates while maintaining lower FP rates than EDiR candidates, underscoring its accuracy and confidence in decision-making. The variability in the ChatGPT’s responses was minimal, indicating a high level of reliability in handling similar types of questions consistently.

The use of artificial intelligence (AI) chatbots in solving medical tasks has garnered increasing attention due to their improved precision, expanding knowledge base, increased computational power, and innovative AI algorithms. ChatGPT-4 has demonstrated a high level of accuracy in solving medical examination questions across various specialties. ChatGPT can achieve up to 87% accuracy on multiple-choice questions related to medical licensing exams including subspecialties in neurology, nephrology, radiology, and general medicine [8, 10–13].

In medical imaging, the use of ChatGPT has been evaluated using test questions from the Canadian Royal College and American Board of Radiology examinations, which followed the SBA format with one correct answer and three distractors [8]. At that time, ChatGPT-3.5 was the latest AI language model and correctly answered 69% of the 150 questions. Its performance was notably better in low-order questions (recall or understanding) achieving an accuracy of 84%, compared to high-order thinking questions, where it had a 60% success rate. In comparison, the average score of 82% in our analysis can be considered much better, as the EDiR exam consists of MRQs with one to four correct answers out of five possible answers, and incorrect selections are penalized. Apprehension about penalization may be the primary reason candidates choose on average fewer answers than ChatGPT, avoiding marking responses where their confidence is lower. Compared with the performance of the ChatGPT-4 in the Japanese Radiology Board Examination, where it correctly answered 80% of the lower-order SBA questions, its accuracy in the EDiR MRQs was higher [14]. While GPT-4o demonstrated superior accuracy, this advantage arises from its pre-training on extensive datasets, contrasting with the focused, experience-based knowledge of EDiR candidates. The disparity is further accentuated by the MRQ format of this part of the EDiR examination. However, the clinical value of this broad theoretical knowledge, which lacks deeper clinical expertise in routine practice, remains uncertain. For this reason, the EDiR examination also includes sections with short cases and evaluations of clinically oriented reasoning, which were not assessed in this study as they rely on visual prompts.

Questions with image prompts were not included in this study. Our preliminary research indicated that chatbots including ChatGPT struggle with image-based questions, even when presented with a single image. As demonstrated by Brin et al, while ChatGPT-4 correctly identified the imaging modality, it accurately determined the anatomical region in 87% of cases and the pathology in only 35% [15]. Their study used single images, unlike full series as they are also available for computed tomography (CT) and magnetic resonance imaging in the EDiR examination. The inherent limitation of being a text-based LLM constraints its ability to analyze and interpret visual data, which is crucial in radiology where image interpretation is fundamental [16, 17]. This limitation also explains why high-order thinking questions were not analyzed, as they rely on medical imaging, which the model cannot process directly [16].

It is unlikely that future iterations of ChatGPT may address these hurdles because the interpretation of radiology images (and their sets) requires large-scale training on annotated medical data specific to each imaging modality. It has therefore been suggested that ChatGPT’s potential in radiology may focus on regenerating radiology reports for clarity, conciseness, ease of understanding, or extracting data from reports for large databases [6, 7].

In a comparative analysis, ChatGPT-4 significantly outperformed its predecessor, ChatGPT-3.5, particularly in questions requiring advanced interpretation and clinical reasoning, achieving higher accuracy rates in non-image-based questions [12, 18]. This evolution in performance highlights the advancements in AI models, with newer iterations such as ChatGPT-4 showing improved capabilities in handling complex medical queries. Previous research on earlier versions of these models, such as ChatGPT-3.5, in an FRCR2A-style mock examination featuring single-best-answer (SBA) questions and clinical case vignettes, revealed that medical residents outperformed Bard, Bing, and ChatGPT-3.5. However, this may no longer hold true given the rapid progress of AI technologies [19].

The ongoing evolution of LLMs suggests a competitive landscape among AI models, with each iteration striving to improve accuracy and applicability including in medical education and practice. Although LLMs exhibit superior performance in specific text-based recall tasks, they remain far from being comparable to subspecialty-trained radiologists in clinical practice and perform poorly in image-based decision-making skills [20]. Future studies should assess how LLMs perform in complex clinical reasoning to provide a more comprehensive evaluation of their capabilities.

The current limitations of LLMs underscore the need for continued development and human oversight. Both LLMs and AI for medical image analysis should inspire a shift in radiologist training placing greater emphasis on clinical reasoning, integration of medical reports, and multimodality imaging rather than relying heavily on basic factual knowledge.

## Study limitations

This study has several limitations. First, we used only text-based low-order thinking questions without visual prompts, limiting the assessment of ChatGPT’s performance in image-based tasks, which are crucial in fields such as radiology. Second, we evaluated only one LLM that was selected based on our pretest analysis and literature review, which may not reflect the capabilities of other models. Third, the relatively small number of questions per subspecialty restricted a deeper exploration of performance differences across various fields. Fourth, the demographic data of EDiR participants were not collected to maintain confidentiality. Finally, although students were instructed to rely exclusively on ChatGPT’s responses, they may have occasionally applied their own strategies (evidenced by the low dispersion of test scores), when the model provided unclear or incomplete answers.

## Conclusions

In solving low-order thinking factual EDiR questions, ChatGPT-4 significantly outperformed EDiR candidates (radiologists), particularly excelling in identifying correct responses. The variability in ChatGPT’s responses was minimal, demonstrating its reliability in handling similar questions. These results demonstrate GPT-4o’s potential in answering text-based radiology questions, but further research is needed to assess its performance across different question formats and candidate populations.

## Data Availability

The data supporting the findings of this study are available from the corresponding author upon reasonable request, subject to adherence to ethical guidelines and data-sharing policies.

## Abbreviations

AI: Artificial intelligence
B2: Upper intermediate English proficiency level
C1: Advanced English proficiency level
C2: Proficient English proficiency level
EDiR: European diploma in radiology
EUR: Euro
FN: False negative
FP: False positive
GDPR: General data protection regulation
GPT: Generative pre-trained transformer
LLM: Large language model
MRQ: Multiple response question
SBA: Single best answer
SD: Standard deviation
TN: True negative
TP: True positive
